# Modulating N-Methyl D-Aspartate Receptors to Enhance Learning of Safety Memories in a Rodent Model of Exposure Therapy

**DOI:** 10.1192/bjo.2024.703

**Published:** 2024-08-01

**Authors:** Alice Thursby, Emily Sherman, Emma Cahill

**Affiliations:** 1 University of Bristol, Bristol, United Kingdom; 2 University of Cambridge, Cambridge, United Kingdom

## Abstract

**Aims:**

Overactive negative memories are thought to contribute to the core symptoms of psychiatric conditions such as anxiety disorders or post-traumatic stress disorder (PTSD). For talking therapies, such as exposure therapy, there are high rates of relapse demonstrating the necessity for innovative new treatments. It is thought that enhancing the ability to extinguish fear responses to the reactivation of these memories in patients with pharmacological adjunct treatments will enhance the efficacy of interventions.

N-methyl D-aspartate receptors (NMDARs) regulate the process of memory formation and consolidation. It is hypothesised that increasing the function of NMDARs would augment the consolidation of safety learning, during treatment sessions. NMDARs require the co-agonists glycine or d-serine to function. Bitopertin, a GlyT-1 inhibitor, increases the availability of glycine. Bitopertin has been studied in the context of schizophrenia, and therefore has been demonstrated to be safe for use in humans. In this preclinical study, we aim to determine if bitopertin can enhance safety learning, so-called extinction, in rodent models.

**Methods:**

24 Lister Hooded rats (male, n = 12) will undergo aversive Pavlovian conditioning to form an associative memory. Rats will then be administered with saline or bitopertin systemically, prior to a session to extinguish fear responses. The strength of the extinction of responses will be measured the following day with a rapid re-acquisition test.

**Results:**

This study is being carried out as part of an intercalated master's degree, so the final results will be available in spring 2024. Given pilot data, it is expected that we will observe that the rats administered with bitopertin exhibit lower levels of fear responses on the rapid reacquisition test than the rats administered with saline. We do not predict any sex difference in responses. This would demonstrate bitopertin has the potential to enhance and safety memory consolidation in rats.

**Conclusion:**

This is an exciting area of research for which results could provide a break-through in improving talking therapies and adjunct treatments offered to patients with anxiety disorders. Negative results would be informative as this allows neurobiologists to refine the search for a pharmacological agent which could be used as a cognitive enhancer in this manner.

